# The MOBCART (magnetic resonance observation of bone and cartilage repair tissue) add-on score

**DOI:** 10.1186/s13244-026-02404-3

**Published:** 2026-09-29

**Authors:** Felix R. M. Koenig, Markus Schreiner, Pavol Szomolanyi, Vladimir Juras, Marcus Raudner, Philipp Sorgo, Gregor Wollner, Giuseppe Filardo, Carl S. Winalski, Ali Guermazi, William D. Bugbee, Siegfried Trattnig

**Affiliations:** 1 High-Field MR Center, Department of Biomedical Imaging and Image-Guided Therapy, Medical University of Vienna, Vienna, Austria; 2 Department of Biomedical Imaging and Image-Guided Therapy, Medical University of Vienna, Vienna, Austria; 3 Department of Orthopedics and Trauma Surgery, Medical University of Vienna, Vienna, Austria; 4 Institute of Measurement Science, Slovak Academy of Sciences, Bratislava, Slovakia; 5 Faculty of Biomedical Sciences, Università della Svizzera Italiana, Lugano, Switzerland; 6 Service of Orthopaedic and Traumatology, Department of Surgery, EOC, Lugano, Switzerland; 7 Department of Diagnostic Radiology, Program of Advanced Musculoskeletal Imaging, Cleveland Clinic, Cleveland, OH USA; 8 Department of Biomedical Engineering, Lerner Research Institute, Cleveland Clinic, Cleveland, OH USA; 9 Department of Radiology, Chobanian & Avedisian School of Medicine, Boston University, Boston, MA USA; 10 Department of Radiology, Boston VA Healthcare System, West Roxbury, MA USA; 11 Department of Orthopaedic Surgery, Scripps Clinic, La Jolla, CA USA; 12 Austrian Cluster for Tissue Regeneration, Vienna, Austria; 13 Karl Landsteiner Institute for Clinical Molecular MR in the Musculoskeletal System, Vienna, Austria

**Keywords:** Magnetic resonance imaging, Knee, Cartilage, Bone and bones, Reproducibility of results

## Abstract

**Objectives:**

To introduce the magnetic resonance observation of bone and cartilage repair tissue (MOBCART) bone score as a dedicated add-on to the established MOCART 2.0 knee score, and to evaluate its reproducibility for MRI-based assessment of the osseous component after osteochondral reconstruction of the knee.

**Materials and methods:**

Thirty-two postoperative knee MRI examinations from 23 patients were scored using the three-domain MOBCART add-on (Integration, Normalization, Cysts; 0–50 points) by four independent readers (expert musculoskeletal radiologist, expert orthopedic cartilage specialist, radiology resident, orthopedic resident). Inter- and intrarater reliability were assessed using linear weighted kappa for individual ordinal domains and intraclass correlation coefficients (ICC; two-way mixed, absolute agreement, single measures) for the total score. Two readers repeated scoring for intrarater reliability.

**Results:**

Interrater reliability across all four readers was moderate-to-good for the total score (ICC(A,1) = 0.72) and substantial-to-almost perfect for domain scores (κ = 0.71–0.82). Expert-reader agreement was good for the total score (ICC(A,1) = 0.90) and almost perfect for all domains (κ = 0.93–0.95). Intrarater reliability was excellent for Reader 1 (ICC(A,1) = 0.98) and good for Reader 4 (ICC(A,1) = 0.85).

**Conclusions:**

The MOBCART add-on score is a reproducible, bone-focused supplement to the MOCART 2.0 knee score that enables standardized MRI reporting of osseous features after osteochondral reconstruction.

**Key Points:**

- **Question** Can a dedicated MRI add-on score to MOCART 2.0 reliably assess the osseous component after osteochondral knee repair across readers of varying experience?
- **Findings** The MOBCART add-on showed moderate-to-good interrater reliability across mixed-experience readers, good agreement between experts, and high test–retest reliability for both experienced and resident readers.
- **Critical relevance statement** As a complement to the MOCART 2.0 knee score, the MOBCART add-on score may facilitate standardized reporting of the osseous compartment after osteochondral reconstruction and enable objective comparison of bone-related imaging outcomes across procedures and studies.

**Graphical Abstract:**

**Figure:**
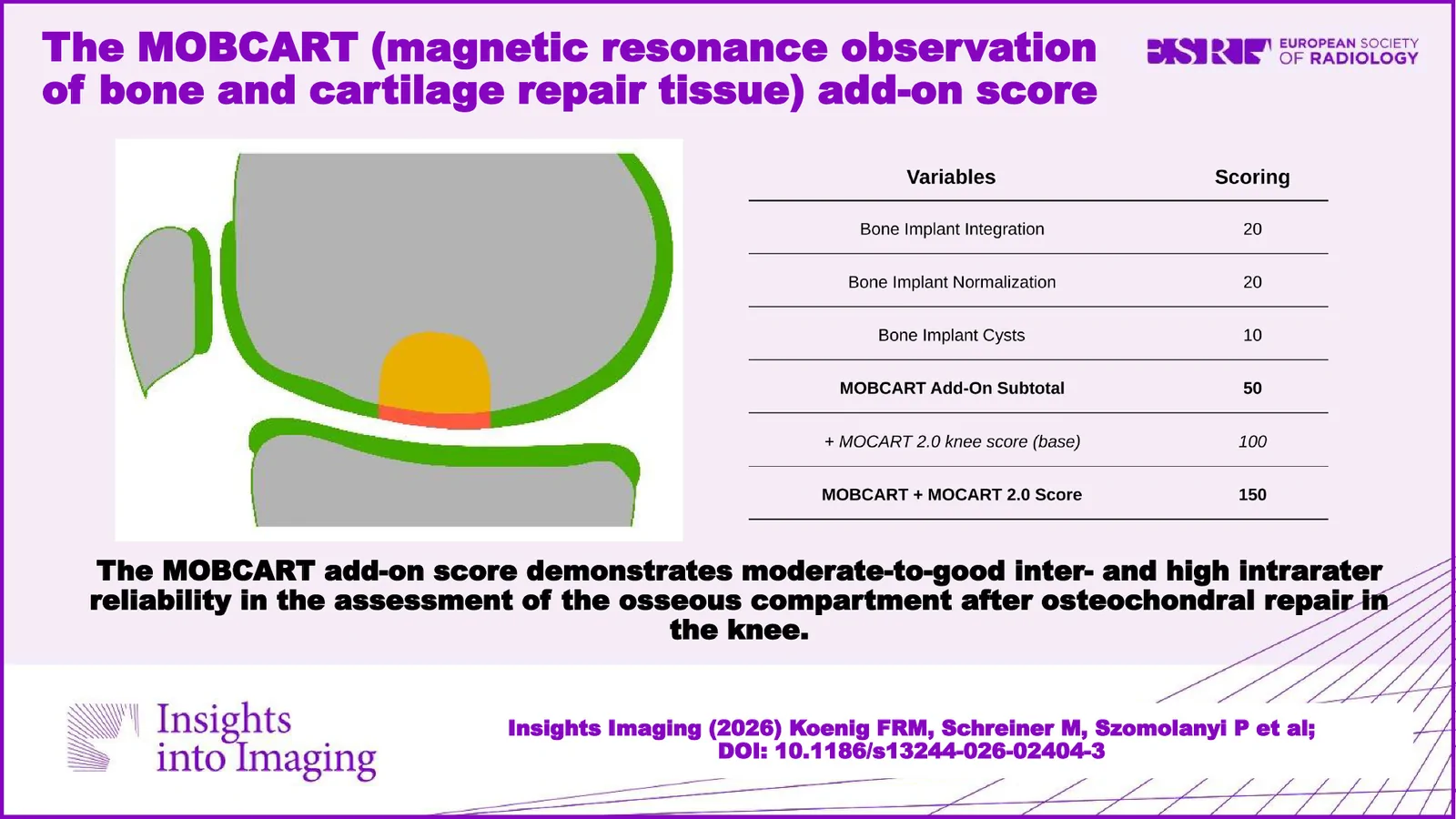

## Introduction

The evaluation of subchondral bone quality is critical to understanding the progression and success of osteochondral repair procedures [1]. In the knee joint, where biomechanical demands are high, the integrity and remodeling of the bone component significantly influence long-term outcomes.

Magnetic resonance imaging (MRI) has emerged as a powerful, noninvasive modality for evaluating osteochondral repair. It enables detailed assessment of the bone–cartilage interface, subchondral architecture, and marrow signal, offering insights beyond what clinical examination or radiographs can provide [2, 3]. Recent years have seen increased use of MRI in conjunction with clinical outcome measures to assess cartilage repair procedures in the knee [4].

To support standardized reporting and follow-up, Trattnig et al developed the MOCART (magnetic resonance observation of cartilage repair tissue) score, which has since become a widely used tool for evaluating cartilage repair [5]. Its successor, the MOCART 2.0 knee score, further improved reproducibility and comparability across time points and surgical techniques [6, 7]. However, the MOCART 2.0 score, while improved over its predecessor for the assessment of cartilage repair, does not fully or specifically evaluate the bone component in osteochondral repair [4]. The MOCART 2.0 knee score was designed primarily to assess the quality of cartilage repair tissue, and although it includes some evaluation of subchondral changes (such as edema-like marrow signals and cysts), its focus remains on the cartilage layer [7].

To address this gap, we developed the magnetic resonance observation of bone and cartilage repair tissue (MOBCART) add-on score—a dedicated, MRI-based supplement to MOCART 2.0—designed to systematically evaluate the bone component in osteochondral repair of the knee. MOBCART complements MOCART 2.0 by providing a structured assessment of bone components that are not adequately captured by cartilage-focused scores alone. The aim of this study was to introduce the MOBCART add-on score and to evaluate its reproducibility for MRI-based assessment of the osseous component after osteochondral reconstruction of the knee.

## Materials and methods

This retrospective study was conducted in accordance with the Declaration of Helsinki and was approved by the local ethics committee (EK 1793/2025); the requirement for informed consent was waived due to the retrospective design. We retrospectively identified patients who had undergone surgical osteochondral treatment of the knee at our tertiary referral center for musculoskeletal imaging and cartilage repair surgery, with at least one postoperative MRI follow-up examination acquired between 2011 and 2025.

Three osteochondral repair procedures were included: osteochondral autograft transfer (OATS) [8], refixation of an osteochondral fragment using a bone screw (Shark Screw®), and cell-free osteochondral scaffold implantation (MaioRegen®, Finceramica Faenza S.p.A., Faenza, Italy) [9, 10]. Cases were intentionally selected to cover a broad spectrum of postoperative osseous appearances, including different follow-up intervals, cases with and without postoperative complications, and varying implant sizes. The primary objective was reliability testing of MOBCART domains across postoperative osseous appearances rather than technique comparison.

### MRI acquisition

All examinations were performed on 3-T MR systems (MAGNETOM Tim Trio, MAGNETOM Verio, MAGNETOM Prisma; Siemens Healthineers, Erlangen, Germany) using a dedicated knee coil. As examinations were obtained as part of routine postoperative clinical follow-up, sequence parameters varied slightly between patients. The protocol was aligned with sequence recommendations established for the MOCART 2.0 framework and included three-plane localizers; sagittal non-fat-saturated proton density-weighted turbo spin echo (PDw TSE); sagittal fat-saturated PDw TSE; sagittal T1-weighted TSE; and coronal fat-saturated PDw TSE. For patellofemoral repair sites, an additional axial fat-saturated PDw TSE was acquired, with the same parameters as for the Cor PDw TSE fs. See a representative MRI protocol in Table 1. All included examinations contained the full indicated postoperative cartilage repair protocol; cases lacking any of the required sequences (e.g., due to motion artifacts or incomplete acquisitions) were excluded from analysis.

**Table 1:** Representative 3-T MRI protocol used for osseous MOBCART add-on assessment

	Example parameters for a 3-T knee protocol			
	Sag PDw TSE	Sag PDw TSE fs	Sag T1w TSE	Cor PDw TSE fs
Coil	8-ch knee	8-ch knee	8-ch knee	8-ch knee
TE (ms)	37	42	12	27
TR (ms) ± deviation	2000 ± 10%	3090 ± 10%	600	4250
Flip angle (°)	90	90	90	90
Fat suppression	No	Yes	No	Yes
FOV (mm)	120	160	150	150
In-plane resolution (mm)	0.31 × 0.37	0.42	0.33	0.39
Acq. matrix	384	384	448	384
Slices	19	25	25	25
Slice thickness (mm)	2	3	3	3
Interslice gap (%)	10	20	20	10
Slice orientation	Sagittal	Sagittal	Sagittal	Coronal
Acquisition time (TA)	03:20	04:06	02:48	03:29

The flip angle reported is the excitation pulse. In-plane resolution is given as frequency × phase pixel size (mm) when asymmetric, or as a single value when isotropic

*Sag PDw TSE* sagittal proton density-weighted turbo spin echo, *Sag PDw TSE fs* sagittal proton density-weighted turbo spin echo, fat-saturated, *Sag T1w TSE* sagittal T1-weighted turbo spin echo, *Cor PDw TSE fs* coronal proton density-weighted turbo spin echo, fat-saturated, *FOV* field of view

For MOBCART scoring, all acquired sequences were used to analyze the osseous component, including graft–host interface integrity, bone marrow pattern/normalization, cysts, trabecular architecture, and structural bone defects. Fat-suppressed sequences were used for assessment of bone marrow edema-like signal alterations and interface-related signal changes, whereas non-fat-saturated PDw/T1-weighted sequences supported evaluation of trabecular architecture, subchondral lamina integrity, and osseous integration. A high in-plane resolution (≤ 0.3 mm) was targeted to enable accurate evaluation of the graft–host interface and subtle structural changes. Cartilage scoring (MOCART 2.0 cartilage domains) was not performed in this study.

### Development and variables of the MOBCART add-on score

The MOBCART add-on score was developed as an osseous-focused extension to the MOCART 2.0 knee score through an iterative expert exchange among cartilage repair specialists involved in this study (radiologists C.W., F.K., A.G., S.T.; orthopedic surgeons M.S., G.F., W.B.). The aim was to provide a standardized MRI-based assessment of the osseous component after osteochondral and composite repair while ensuring strict separation from existing MOCART 2.0 variables to avoid double scoring.

The final MOBCART add-on consists of three bone-specific domains and provides up to 50 additional points, resulting in a combined maximum of 150 points when applied together with the 0–100 point MOCART 2.0 knee score. The score is intended osteochondral repair procedures such as OATS, refixation of osteochondral fragments, and osteochondral scaffold implantation. To standardize scoring, a color-coded atlas was developed for each value of each MOBCART variable and is provided as Supplementary Material. Grading and point scale of each subdomain are illustrated in Table 2.

**Table 2:** MOBCART add-on score: osseous implant assessment—grading and point scale

MOBCART add-on score—bone and cartilage repair tissue assessment			
Category		Description	Points
1	Bone implant integration		Max 20
	A	Complete integration	20
	B	Circumferential or partial demarcating borderline < 2 mm	15
	C	Split-like bony defect (lateral graft–host interface) < 2 mm	10
	D	Split-like bony defect (basal graft–host interface) < 2 mm	5
	E	Integration defect > 2 mm (lateral and/or basal graft–host interface) and/or collapsed graft surface	0
2	Bone implant normalization		Max 20
	A	Bone-like marrow signal throughout the implant	20
	B	Edema-like signal throughout the implant (≥ 30% of implant volume, trabecular bone lines visible)	15
	C	Majority (90–50%) shows bone normalization	10
	D	Minority (49–10%) shows bone normalization	5
	E	No visible bone normalization (< 10% of implant/scaffold volume)	0
3	Bone implant cysts		Max 10
	A	No implant cysts	10
	B	Implant cysts ≤ 5 mm	5
	C	Implant cysts > 5 mm	0
	Total score		50

**Fig. 1:**
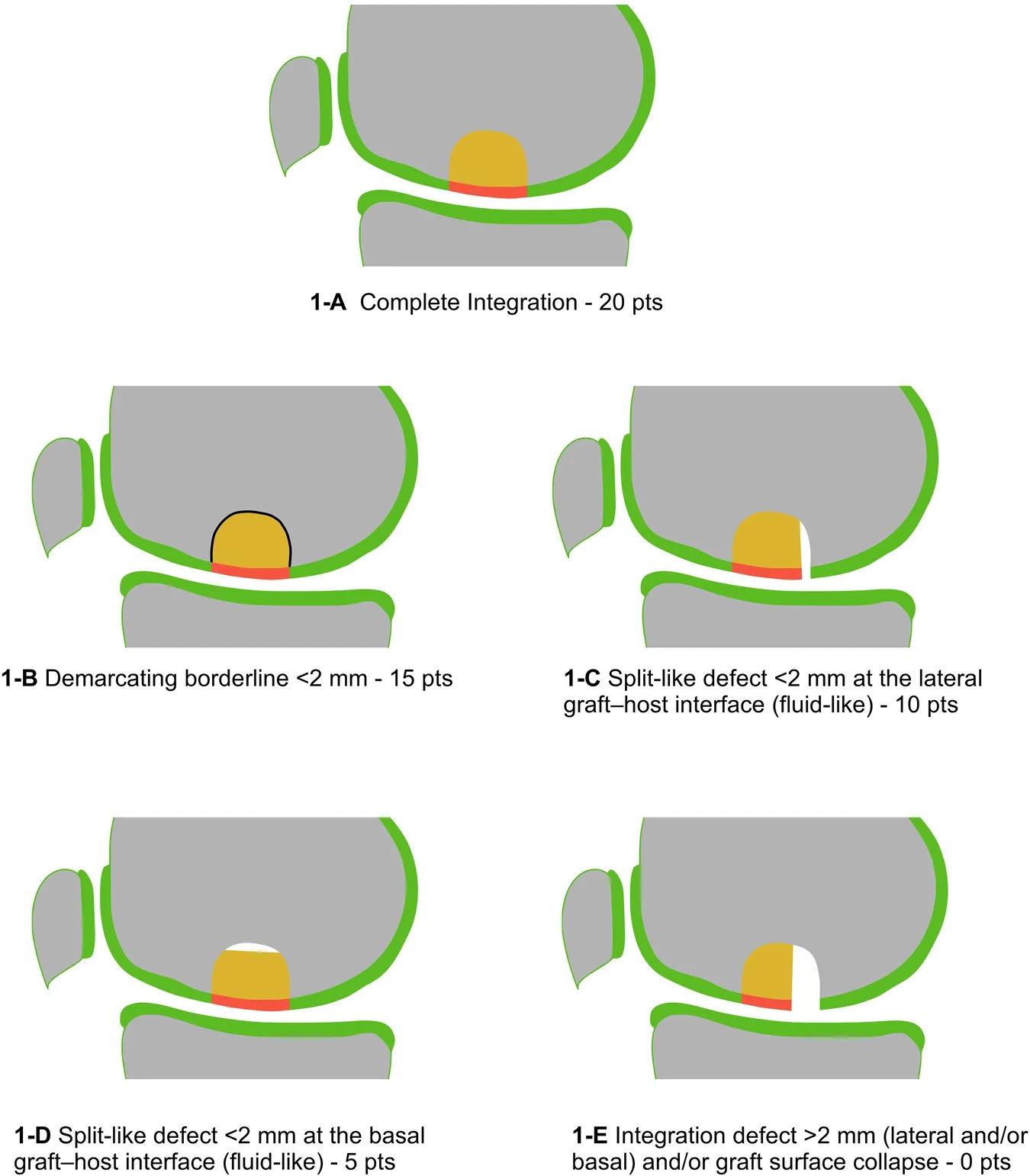
MOBCART domain—bone implant integration. Representative examples of the five-grade bone implant integration domain, assessing osseous integration at the graft/scaffold–host bone interface (the worst interface determines the grade). **A** Complete integration (20 points). **B** Circumferential or partial demarcating borderline < 2 mm (15 points). **C** Split-like bony defect < 2 mm at the lateral graft–host interface, fluid-like signal (10 points). **D** Split-like bony defect < 2 mm at the basal graft–host interface, fluid-like signal (5 points). **E** Integration defect > 2 mm (lateral and/or basal graft–host interface) and/or collapsed graft surface (0 points)

**Bone implant integration** assesses the degree of osseous integration of the transplant/scaffold to surrounding native bone at the graft–host interface, focusing on continuity rather than signal characteristics (Fig. 1). The worst interface determines the grade (lateral/circumferential vs basal/deep). Integration is graded as complete when no demarcation is discernible (Fig. 1A). A circumferential or partial demarcating borderline < 2 mm is defined as a thin interface without a true gap (Fig. 1B). Split-like bony defects < 2 mm are graded by location—lateral/circumferential (Fig. 1C) versus basal/deep (Fig. 1D)—and demonstrate fluid-like signal on PD/T2-weighted images. An integration defect > 2 mm and/or collapsed graft surface represents structural failure (Fig. 1E).

**Fig. 2:**
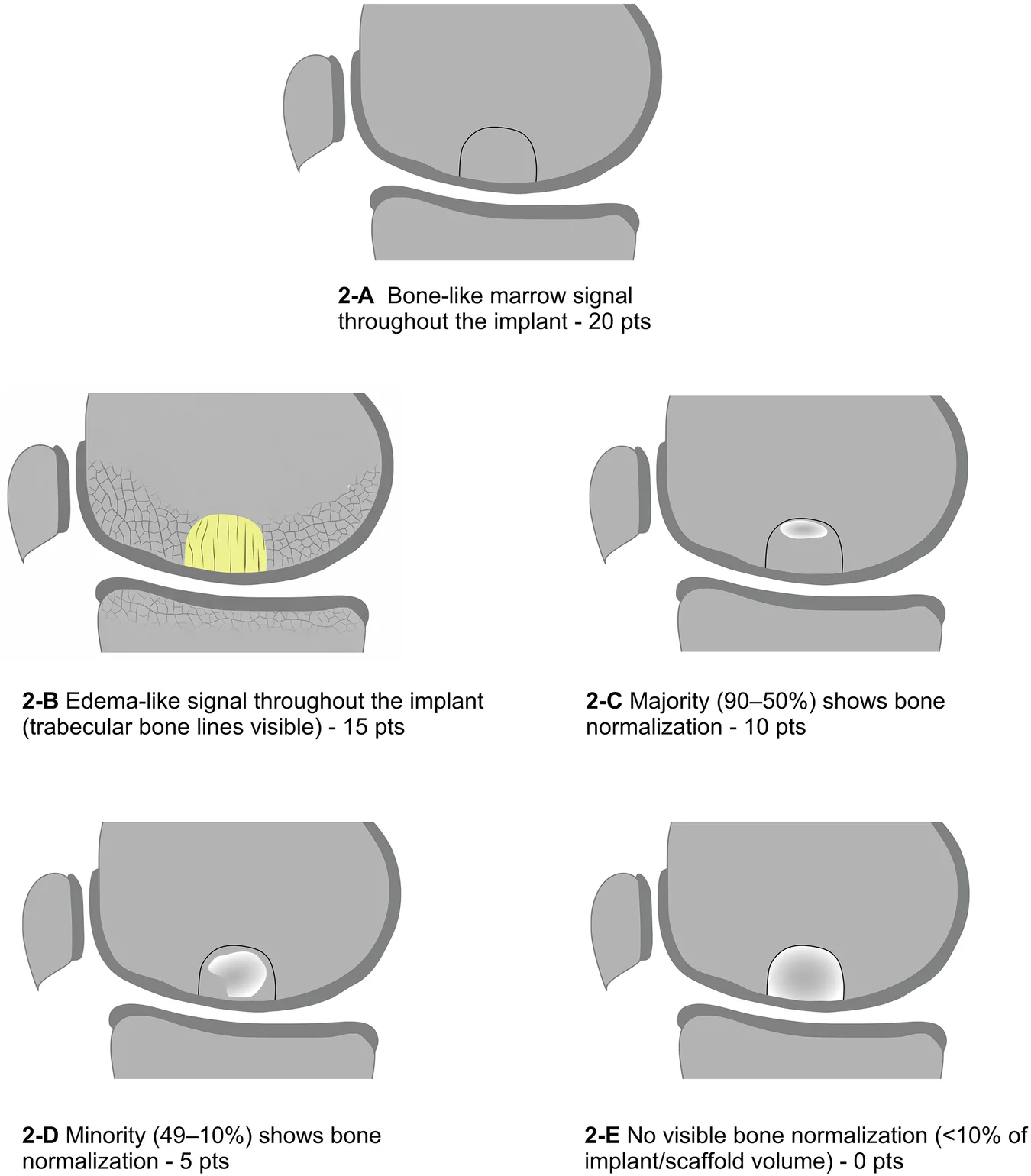
MOBCART domain—bone implant normalization. Representative examples of the five-grade bone implant normalization domain, assessing structural remodeling/normalization of the implanted osseous component relative to surrounding native bone. **A** Bone-like marrow signal throughout the implant (20 points). **B** Edema-like signal throughout the implant with trabecular bone lines still visible (15 points). **C** Majority (90–50%) of the implant shows bone normalization (10 points). **D** Minority (49–10%) of the implant shows bone normalization (5 points). **E** Novisible bone normalization (< 10% of implant/scaffold volume) (0 points)

**Bone implant normalization** assesses structural normalization of the implanted osseous component compared with surrounding native bone, focusing on trabecular organization rather than focal subchondral bone marrow edema (the latter being captured by MOCART 2.0). Trabecular bone lines are best assessed on non-fat-saturated PD-weighted TSE sequences; absence of trabecular lines is interpreted as lack of bone normalization (Fig. 2). Normalization is graded from bone-like marrow signal throughout the implant (Fig. 2A) to no visible bone normalization (< 10% of implant volume) (Fig. 2E). Edema-like signal throughout the implant is defined as ≥ 30% of the implant volume with trabecular lines still visible (Fig. 2B). Partial normalization is graded as majority (90–50%) (Fig. 2C) or minority (49–10%) (Fig. 2D).

**Fig. 3:**
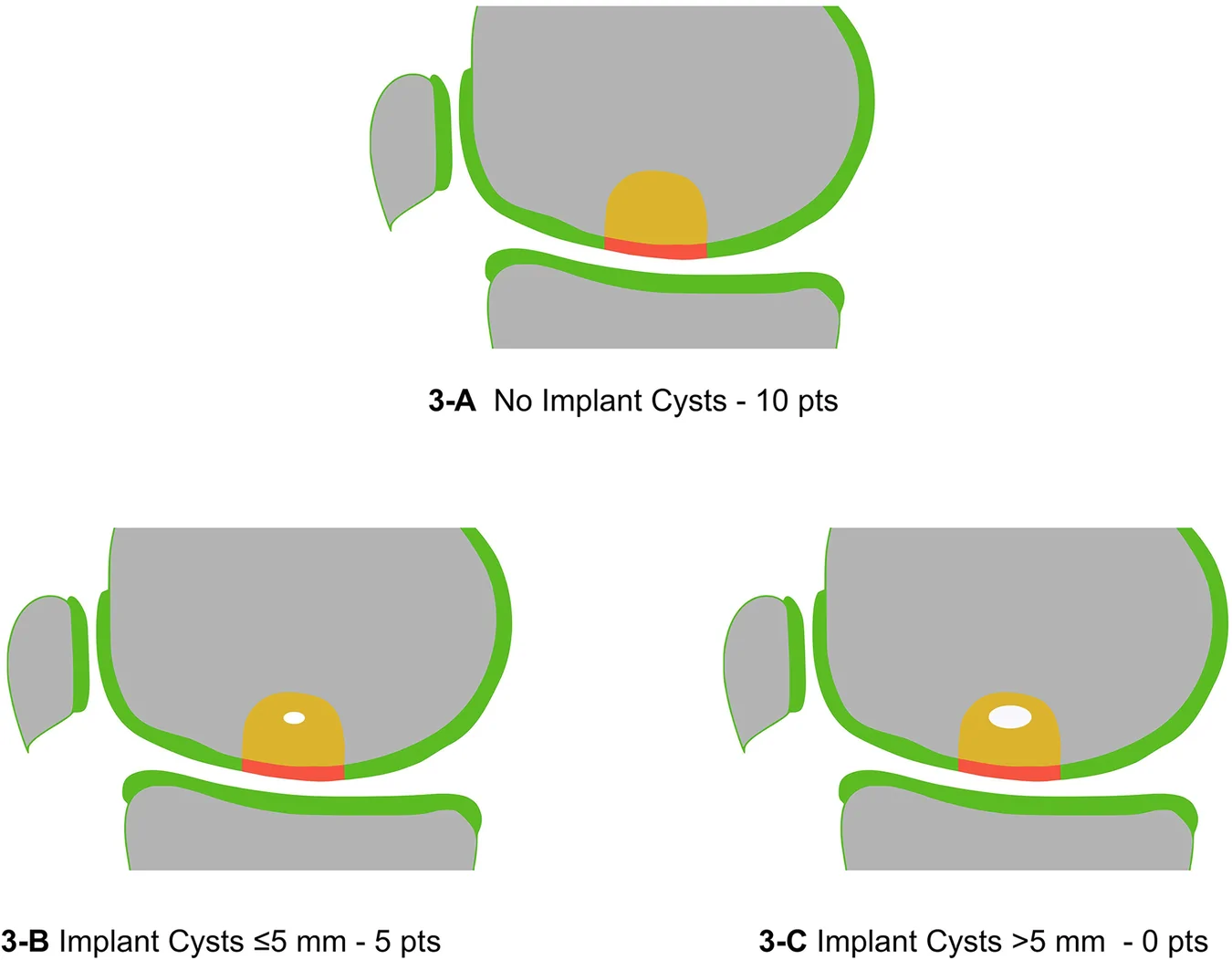
MOBCART domain—bone implant cysts. Representative examples of the three-grade bone implant cysts domain, assessing intra-implant cysts located within the osseous component and/or in direct contact with the implant–host bone interface (combined diameter is used if multiple cysts are present). **A** No implant cysts (10 points). **B** Implant cysts ≤ 5 mm (5 points). **C** Implant cysts > 5 mm (0 points)

**Bone implant cysts** assess intra-implant cysts within the osseous component in direct contact with the implant–host bone interface and/or located within the implant (Fig. 3). Cysts are defined as rounded/ovoid, well-demarcated, fluid-like cavities and must be distinguished from split-like interface defects, which present as linear/cleft-like separations rather than circumscribed cavities. If multiple cysts are present, the combined diameter is the sum of maximal diameters. Grading ranges from no implant cysts (10 points) to cysts ≤ 5 mm (5 points) and cysts > 5 mm (0 points) (Fig. 3A–C).

If multiple repair sites were present in one knee, the lowest-scoring implant determined the final MOBCART add-on score, reflecting the clinically dominant “weakest link” principle. A strict conceptual boundary to MOCART 2.0 was maintained: MOCART 2.0 incorporates subchondral changes including edema-like marrow signal, subchondral cysts, and bony defect/overgrowth in the repair region, whereas MOBCART focuses on osseous incorporation and remodeling within the implanted bone compartment and on cysts inside the transplant or at the graft–host osseous interface (rather than superficial subchondral cysts adjacent to the cartilage repair zone, which are already represented in MOCART 2.0).

### Image analysis and readers

Image analysis was performed on a picture archiving and communication system (PACS) workstation (IMPAX EE R20, Agfa Healthcare N.V., Mortsel, Belgium) by four independent readers. Prior to formal scoring, five exemplary cases were jointly reviewed to familiarize readers with the atlas. All cases were assessed independently in randomized reading order with blinding to patient identity, surgery timepoint, and clinical outcome; clinical information was limited to the operated site and repair technique. The reader panel comprised: (1) a cartilage-focused musculoskeletal radiologist with > 30 years of experience (Reader 1); (2) an orthopedic cartilage specialist with > 10 years of experience (Reader 2); (3) a radiology resident without prior expertise in cartilage repair MRI (Reader 3); and (4) an orthopedic resident without prior expertise in cartilage repair MRI (Reader 4). Interrater reliability was calculated based on the first reading of all four readers. To assess intrarater reliability, Reader 1 and Reader 4 repeated the complete evaluation after a 4-week interval with a re-randomized case order to minimize recall bias, with all readers having access to the supplementary schematic MOBCART atlas throughout scoring.

### Statistical analysis

All statistical calculations were performed with IBM SPSS Statistics for Windows, version 31 (IBM, Armonk, NY, USA). Metric data are described as mean ± standard deviation. Linear weighted kappa statistics with 95% confidence intervals (CIs) were calculated as an index of inter- and intrarater reliability for each ordinal MOBCART scoring domain and interpreted according to Landis and Koch: κ ≤ 0.20 indicating poor, 0.21–0.40 fair, 0.41–0.60 moderate, 0.61–0.80 substantial, and ≥ 0.81 almost perfect agreement [11]. For interrater reliability of ordinal domain scores with four readers, pairwise linear weighted Cohen’s κ was calculated for each rater pair and reported as the mean pairwise κ. Inter- and intrarater reliability of the overall MOBCART total score was assessed using the intraclass correlation coefficient (two-way mixed-effects model, absolute agreement, single measures [ICC(A,1)]) with 95% CIs and interpreted according to Koo and Li: ICC < 0.50 indicating poor, 0.50–0.75 moderate, 0.75–0.90 good, and > 0.90 excellent agreement [12].

## Results

### Patients

A total of 32 postoperative knee MRI examinations from 23 patients were included (15 OATS, 2 Shark Screw refixations, 15 after cell-free osteochondral scaffold implantation). Mean patient age at the time of surgery was 33.7 years (range 16.9–52.9); 18/23 (78%) patients were male and 5/23 (22%) female.

Defect/implant size was obtained from the surgical report or, if not reported, measured on the first available postoperative MRI. Dimensions were recorded as maximal lesion extent × osteochondral defect depth (cm): anterior–posterior (AP) × depth for femoral/tibial lesions and proximal–distal × depth for patellar lesions. Overall, the mean linear dimensions were 1.40 × 0.96 cm (extent 0.80–2.80 cm; depth 0.50–1.40 cm). The corresponding mean defect area was 1.34 cm² (median 1.17 cm²; range 0.40–2.80 cm²), and mean depth was 0.96 cm (median 0.90 cm; range 0.50–1.40 cm). Lesions were most commonly located in the medial femoral condyle (53%), followed by the lateral femoral condyle (38%), with fewer cases in the trochlea (6%) and patella (3%).

In the OATS/Shark Screw group, mean defect dimensions were 1.12 × 1.13 cm; in the MaioRegen group, 1.75 × 0.76 cm. In the OATS/Shark Screw group, the mean number of cylinders/plugs was 1.41 (range 1–2), with 10/17 (59%) single-plug and 7/17 (41%) two-plug reconstructions; for cases with multiple plugs, the largest plug was used for size description.

Across all MRI examinations, the mean time since surgery was 18.7 months (median 12.9; range 0.7–81.7).

### Inter- and intrarater reliability

Interrater reliability of the MOBCART add-on score differed by domain and reader experience. Across all four readers, mean linear weighted κ indicated substantial agreement for Bone Implant Integration (κ = 0.71, 95% CI 0.51–0.87), almost perfect agreement for Bone Implant Normalization (κ = 0.82, 95% CI 0.69–0.91), and substantial-to-almost perfect agreement for Bone Implant Cysts (κ = 0.80, 95% CI 0.65–0.92). Agreement was highest between the expert readers (R1–R2), with almost perfect agreement across all domains (Integration κ = 0.93, 95% CI 0.77–1.00; Normalization κ = 0.95, 95% CI 0.82–1.00; Cysts κ = 0.95, 95% CI 0.81–1.00). Reliability of the overall MOBCART total score was moderate-to-good across all four readers (ICC(A,1) = 0.72, 95% CI 0.52–0.88) and good when restricted to the expert readers (ICC(A,1) = 0.90, 95% CI 0.65–1.00).

Intrarater (test–retest) reliability was excellent for Reader 1 (Integration κ = 0.94, 95% CI 0.80–1.00; Normalization κ = 0.98, 95% CI 0.93–1.00; Cysts κ = 1.00, 95% CI 1.00–1.00; total score ICC(A,1) = 0.98, 95% CI 0.95–1.00) and moderate-to-excellent for Reader 4 (Integration κ = 0.77, 95% CI 0.55–0.95; Normalization κ = 0.85, 95% CI 0.73–0.94; Cysts κ = 0.74, 95% CI 0.53–0.94; total score ICC(A,1) = 0.85, 95% CI 0.68–0.95) (Table 3).

**Table 3:** Interrater and intrarater reliability of the MOBCART bone add-on score

Variables MOBCART score	Interrater (all 4 readers)		Interrater (experts R1–R2)	
Interrater reliability	ICC/κ	95% CI	ICC/κ	95% CI
Overall (total score)	ICC 0.72	0.52–0.88	ICC 0.90	0.65–1.00
Bone implant integration	κ 0.71	0.51–0.87	κ 0.93	0.77–1.00
Bone implant normalization	κ 0.82	0.69–0.91	κ 0.95	0.82–1.00
Bone implant cysts	κ 0.80	0.65–0.92	κ 0.95	0.81–1.00
**Variables MOBCART score**	**Intrarater (Reader 1)**		**Intrarater (Reader 4)**	
**Intrarater reliability**	**ICC/κ**	**95% CI**	**ICC/κ**	**95% CI**
Overall (total score)	ICC 0.98	0.95–1.00	ICC 0.85	0.68–0.95
Bone implant integration	κ 0.94	0.80–1.00	κ 0.77	0.55–0.95
Bone implant normalization	κ 0.98	0.93–1.00	κ 0.85	0.73–0.94
Bone implant cysts	κ 1.00	1.00–1.00	κ 0.74	0.53–0.94

R1–R4 = readers 1–4 (R1 = expert MSK radiologist; R2 = expert orthopedic cartilage specialist; R3 = radiology resident; R4 = orthopedic resident)

*MOBCART* magnetic resonance observation of bone and cartilage repair tissue, *ICC* intraclass correlation coefficient, *ICC(A,1)* two-way mixed-effects model, absolute agreement, single measures,

*κ* linear weighted Cohen’s kappa, *CI* confidence interval

## Discussion

The MOCART and, more recently, MOCART 2.0 knee scores represent the most widely adopted semiquantitative MRI grading systems for morphological assessment of cartilage repair tissue and serve as the basis for standardized reporting in many centers [4–7, 13]. Building on this established framework, this study introduces the MOBCART bone add-on score as a non-overlapping supplement to MOCART 2.0, dedicated to noninvasive MRI assessment of the osseous component after osteochondral reconstruction of the knee. The score was designed to capture key bone-related features—implant integration, bone remodeling/normalization, and intra-implant cysts—while maintaining strict conceptual separation from MOCART 2.0 domains.

The main finding of this study was that the reproducibility of the MOBCART bone add-on score was high overall but dependent on reader experience and scoring domain. Across all four readers, interrater reliability of the total MOBCART score was moderate-to-good (ICC(A,1) = 0.72), while domain-level agreement ranged from substantial to almost perfect (mean pairwise κ = 0.71 for Bone Implant Integration, 0.82 for Normalization, and 0.80 for Cysts). In contrast, agreement between the two expert readers was good for the total score (ICC(A,1) = 0.90) and almost perfect for all domains (κ = 0.93–0.95), indicating that lower overall interrater estimates were driven primarily by discrepancies involving less experienced raters. Intrarater (test–retest) reliability was high, with excellent stability for the expert reader (ICC(A,1) = 0.98) and good stability for the resident reader (ICC(A,1) = 0.85). Notably, all readers had continuous access to the supplementary schematic MOBCART atlas during scoring; the substantial-to-almost perfect domain-level agreement achieved by the two non-expert readers suggests that this atlas is sufficient for reliable application of the score by less experienced raters. Domain-level repeatability was highest for Normalization and Integration, while Cysts showed the lowest repeatability in the less experienced reader. These differences likely reflect the greater interpretive complexity of graft–host interface assessment and cyst categorization and the heterogeneity of postoperative osseous appearances after osteochondral reconstruction.

A key rationale for including Bone Implant Integration as a core MOBCART domain is the central role of the subchondral bed and graft–host union in osteochondral reconstruction. Gomoll et al emphasized that without support from an intact subchondral bed, treatment of the surface chondral lesion is likely to fail [1]. This concept is supported by prospective imaging evidence: Brown et al used a 6-month CT-based incorporation score after fresh osteochondral allograft (OCA) of the distal femur and reported that a greater degree of osseous incorporation was associated with earlier clinical improvement [14]. Accordingly, MOBCART operationalizes integration by considering circumferential (lateral) versus deep (basal) interface integrity and applying a worst-interface rule, reflecting the clinically dominant “weakest link” concept of osteochondral construct stability.

A second rationale for the Bone Implant Normalization domain is that long-term success of osteochondral reconstruction depends not only on interface union but on restoration of a structurally organized subchondral bone compartment, with incomplete remodeling potentially compromising the quality of the osteochondral unit [1]. Quantitative MRI after matrix-associated autologous chondrocyte implantation (MACI) with autologous bone grafting has shown that early postoperative trabecular microstructure metrics are significantly associated with later morphological joint outcome [15]. MOBCART translates this into routine MRI by using trabecular line continuity and a bone-like marrow pattern as pragmatic surrogates of structural normalization [16], distinct from focal host-bone edema-like changes already covered by MOCART 2.0.

Cystic changes represent another clinically relevant osseous feature after osteochondral repair. Cyst formation has been linked to altered bone remodeling, impaired microarchitecture, and abnormal stress distribution rather than being a purely incidental imaging finding [17]. The MOBCART cyst domain is defined to capture intra-implant/interface-adjacent cysts, thereby avoiding overlap with MOCART 2.0 subchondral cyst definitions adjacent to the cartilage repair zone. Intra-implant cyst formation may represent compromised osseous remodeling at the reconstructed osteochondral unit and warrants structured documentation, although its prognostic relevance remains to be clarified [17, 18].

The MOBCART score was designed to be applicable across two fundamentally different surgical concepts: OATS and cell-free osteochondral scaffold implantation. Despite their biological and biomechanical differences, the score demonstrated moderate-to-good interrater reliability across all four readers, with excellent agreement among expert readers, supporting its robustness for both plug-based transplantation and scaffold-based reconstruction. These procedure families follow distinct postoperative trajectories—slow, prolonged maturation in scaffold-based reconstruction [19–21] versus rapid early structural appearance with later interface- or implant-related complications in plug-based transplantation [8, 22]—strengthening the rationale for a dedicated osseous score that is interpretable across techniques.

Several limitations should be acknowledged. First, this was a retrospective reliability study and did not aim to establish prognostic validity; the relationship between MOBCART domains and patient-reported outcomes remains to be demonstrated in future cohorts with systematic clinical correlation. Second, postoperative MRI examinations were acquired as part of routine clinical follow-up with slightly varying sequence parameters; nevertheless, reliability remained high, suggesting that the score is robust under real-world clinical imaging conditions. Third, we did not perform formal subgroup analyses by surgical technique, and future work should evaluate the metric properties and potential technique-specific behavior of individual domains across a broader range of osteochondral reconstruction procedures, including cell-based repairs with subchondral bone grafting (e.g., sandwich techniques or staged bone grafting followed by subsequent MACI). Fourth, the protocol included a sagittal T1-weighted sequence; applicability with more widely used protocols employing a coronal T1 (e.g., the ESSR Knee MR protocol) was not evaluated and warrants future work. Fifth, the MOBCART add-on was deliberately designed not to overlap with the MOCART 2.0 knee score, and the present study focused on establishing the MOBCART add-on in isolation; future studies will address its application across different cartilage repair techniques and in routine combination with MOCART 2.0. Sixth, osteochondral defect size was characterized by one in-plane axis plus depth; for elongated defects, a two-axis in-plane characterization (e.g., anterior–posterior and transverse) plus depth is recommended for future applications.

In conclusion, the MOBCART bone add-on score provides a structured, reproducible, semiquantitative MRI framework to describe the morphology of the osseous component after osteochondral reconstruction. As a supplement to MOCART 2.0, it may facilitate standardized reporting and enable objective comparison of bone-related morphological outcomes across techniques and studies. Future research should validate MOBCART in larger multicenter cohorts, include external readers, and investigate associations with clinical outcomes and complementary imaging markers.

## Supplementary information

ELECTRONIC SUPPLEMENTARY MATERIAL

## Data Availability

The data underlying this article cannot be shared publicly to protect the privacy of individuals who participated in the study. The data underlying this article will be shared on reasonable request to the corresponding author.

## Abbreviations

CI: Confidence interval
fs: Fat-saturated
ICC: Intraclass correlation coefficient
ICC(A,1): Two-way mixed-effects model, absolute agreement, single measures
MACI: Matrix-associated autologous chondrocyte implantation
MOBCART: Magnetic resonance observation of bone and cartilage repair tissue
MOCART: Magnetic resonance observation of cartilage repair tissue
MRI: Magnetic resonance imaging
OATS: Osteochondral autograft transfer system
PDw: Proton density-weighted
TSE: Turbo spin echo

## References

[1] Gomoll AH, Madry H, Knutsen G et al (2010) The subchondral bone in articular cartilage repair: current problems in the surgical management. Knee Surg Sports Traumatol Arthrosc 18:434–447. 10.1007/s00167-010-1072-x PMC2839476 20130833

[2] Crema MD, Roemer FW, Marra MD et al (2011) Articular cartilage in the knee: current MR imaging techniques and applications in clinical practice and research. Radiographics 31:37–61. 10.1148/rg.311105084 PMC6939857 21257932

[3] Roemer FW, Guermazi A, Trattnig S et al (2014) Whole joint MRI assessment of surgical cartilage repair of the knee: cartilage repair osteoarthritis knee score (CROAKS). Osteoarthritis Cartilage 22:779–799. 10.1016/j.joca.2014.03.014 24685525

[4] Oettl FC, Leuthard L, Brunner M et al (2025) Correlation and comparative evaluation of MOCART and MOCART 2.0 for assessing cartilage repair. Medicina 61:745. 10.3390/medicina61040745 PMC12028356 40283036

[5] Marlovits S, Singer P, Zeller P, Mandl I, Haller J, Trattnig S (2006) Magnetic resonance observation of cartilage repair tissue (MOCART) for the evaluation of autologous chondrocyte transplantation: determination of interobserver variability and correlation to clinical outcome after 2 years. Eur J Radiol 57:16–23. 10.1016/j.ejrad.2005.08.007 16203119

[6] Schreiner MM, Raudner M, Röhrich S et al (2021) Reliability of the MOCART (magnetic resonance observation of cartilage repair tissue) 2.0 knee score for different cartilage repair techniques—a retrospective observational study. Eur Radiol 31:5734–5745. 10.1007/s00330-021-07688-1 PMC8270819 33515088

[7] Schreiner MM, Raudner M, Marlovits S et al (2021) The MOCART (magnetic resonance observation of cartilage repair tissue) 2.0 knee score and atlas. Cartilage 13:571S–587S. 10.1177/1947603519865308 PMC8725373 31422674

[8] Guzman AJ, Dela Rueda T, Rayos Del Sol SM et al (2021) Arthroscopic osteochondral autograft transfer system procedure of the lateral femoral condyle with donor-site backfill using osteochondral allograft plug. Arthrosc Tech 10:e2683. 10.1016/j.eats.2021.08.012 PMC8719136 35004149

[9] D’Ambrosi R, Valli F, De Luca P, Ursino N, Usuelli FG (2019) MaioRegen osteochondral substitute for the treatment of knee defects: a systematic review of the literature. J Clin Med 8:783. 10.3390/jcm8060783 PMC6617307 31159439

[10] De Marziani L, Boffa A, Andriolo L et al (2025) Cell‐free biomimetic osteochondral scaffold for the treatment of knee articular surface lesions: Clinical outcomes differ based on patient and lesion characteristics. Knee Surg Sports Traumatol Arthrosc 33:544–554. 10.1002/ksa.v33.210.1002/ksa.12402 PMC11792099 39101253

[11] Landis JR, Koch GG (1977) The measurement of observer agreement for categorical data. Biometrics 33:159–174. 10.2307/2529310 843571

[12] Koo TK, Li MY (2016) A guideline of selecting and reporting intraclass correlation coefficients for reliability research. J Chiropr Med 15:155–163. 10.1016/j.jcm.2016.02.012 PMC4913118 27330520

[13] Migliorini F, Maffulli N, Eschweiler J, Driessen A, Tingart M, Baroncini A (2021) Reliability of the MOCART score: a systematic review. J Orthop Traumatol 22:39. 10.1186/s10195-021-00603-w PMC8494868 34613499

[14] Brown D, Shirzad K, Lavigne SA, Crawford DC (2011) Osseous integration after fresh osteochondral allograft transplantation to the distal femur: a prospective evaluation using computed tomography. Cartilage 2:337–345. 10.1177/1947603511410418 PMC4297133 26069592

[15] Gersing AS, Holwein C, Suchowierski J et al (2020) Cartilage T2 relaxation times and subchondral trabecular bone parameters predict morphological outcome after matrix-associated autologous chondrocyte implantation with autologous bone grafting. Am J Sports Med. 10.1177/0363546520965987 33200942

[16] Koenig FRM, Janacova V, Schreiner M et al (2026) Routine MRI Signal Intensity as a Surrogate of Subchondral Bone Healing: Validation Against Micro-CT and Histology in an Ovine Model. Diagnostics (Basel) 16:2426. 10.3390/diagnostics16152426 PMC13464844 42587663

[17] Gao L, Cucchiarini M, Madry H (2020) Cyst formation in the subchondral bone following cartilage repair. Clin Transl Med 10:e248. 10.1002/ctm2.248 PMC7733665 33377663

[18] Yang Y, Zhang L (2024) [Research progress of subchondral bone cyst after cartilage repair]. Zhonghua Wai Ke Za Zhi 62:613–618. 10.3760/cma.j.cn112139-20231226-00298 38682634

[19] Di Martino A, Perdisa F, Filardo G et al (2021) Cell-free biomimetic osteochondral scaffold for the treatment of knee lesions: clinical and imaging results at 10-year follow-up. Am J Sports Med 49:2645–2650. 10.1177/03635465211029292 34283948

[20] Brix M, Kaipel M, Kellner R et al (2016) Successful osteoconduction but limited cartilage tissue quality following osteochondral repair by a cell-free multilayered nano-composite scaffold at the knee. Int Orthop 40:625–632. 10.1007/s00264-016-3118-2 26803322

[21] Kaipel M, Trattnig S, Domayer S, Windhager R, Brix M (2016) Clinical and bioimaging results following osteochondral lesion repair using MaioRegen® allograft. Orthop J Sports Med 4:2325967116S00046. 10.1177/2325967116S00046

[22] Solheim E, Hegna J, Inderhaug E (2019) Clinical outcome after mosaicplasty of knee articular cartilage defects of patellofemoral joint versus tibiofemoral joint. J Orthop 18:36–40. 10.1016/j.jor.2019.10.010 PMC7068006 32189881

