# Supplementary material for: The MOBCART (magnetic resonance observation of bone and cartilage repair tissue) add-on score
Source: Insights Imaging. 2026 Sep 29;17:239. doi: 10.1186/s13244-026-02404-3 (PMC13624228; doi:10.1186/s13244-026-02404-3)
Supplement: Supplementary file 1 [file 13244_2026_2404_MOESM1_ESM.pdf]

The MOBCART (Magnetic Resonance Observation of Bone and Cartilage Repair Tissue) add-on score

ELECTRONIC SUPPLEMENTARY MATERIAL: Schematic scoring atlas

This extension to the MOCART (magnetic resonance observation of cartilage repair tissue) 2.0 knee score is designed to specifically assess the osseous component after osteochondral and composite repair. The add-on awards up to 50 additional points in three bone-specific domains (bone implant integration, bone implant normalization, bone implant cysts) to the established MOCART 2.0 knee score (0–100 points), resulting in a combined maximum of 150 points. Cartilage repair tissue and subchondral changes adjacent to the cartilage repair zone remain scored within MOCART 2.0.

The schematic drawings on the following pages illustrate each grade of each domain; panel labels (1-A to 3-C) correspond to Figs. 1–3 of the article. Drawings are shown in sagittal orientation (femoral condyle above, tibial plateau below, patella on the left). The basal/deep graft–host interface therefore lies at the top of the implant, the lateral/circumferential interface along its sides.

Score overview

Domain	Grade	Description	Points
**1**		**Bone implant integration**	**Max 20**
	**A**	Complete integration	20
	**B**	Circumferential or partial demarcating borderline < 2 mm	15
	**C**	Split-like bony defect (lateral graft–host interface) < 2 mm	10
	**D**	Split-like bony defect (basal graft–host interface) < 2 mm	5
	**E**	Integration defect > 2 mm (lateral and/or basal graft–host interface) and/or collapsed graft surface	0
**2**		**Bone implant normalization**	**Max 20**
	**A**	Bone-like marrow signal throughout the implant	20
	**B**	Edema-like signal throughout the implant (≥ 30% of implant volume, trabecular bone lines visible)	15
	**C**	Majority (90–50%) shows bone normalization	10
	**D**	Minority (49–10%) shows bone normalization	5
	**E**	No visible bone normalization (< 10% of implant/scaffold volume)	0
**3**		**Bone implant cysts**	**Max 10**
	**A**	No implant cysts	10
	**B**	Implant cysts ≤ 5 mm	5
	**C**	Implant cysts > 5 mm	0
		**MOBCART add-on total score**	**50**
		+ MOCART 2.0 knee score (cartilage, base score)	100
		**MOCART 2.0 + MOBCART combined maximum**	**150**

Bone Implant Integration – 20 points

Assesses the degree of osseous integration of the transplant/scaffold to the surrounding native bone at the graft–host interface, focusing on continuity of the bone interface and incorporation into the host skeleton rather than signal characteristics. The worst interface determines the grade (lateral/circumferential vs basal/deep). A circumferential or partial demarcating borderline < 2 mm is a thin interface without a true gap. Split-like defects show fluid-like signal (water-isointense) on PD/T2-weighted images, in contrast to a demarcating borderline. An integration defect > 2 mm and/or a collapsed graft surface represents structural failure.

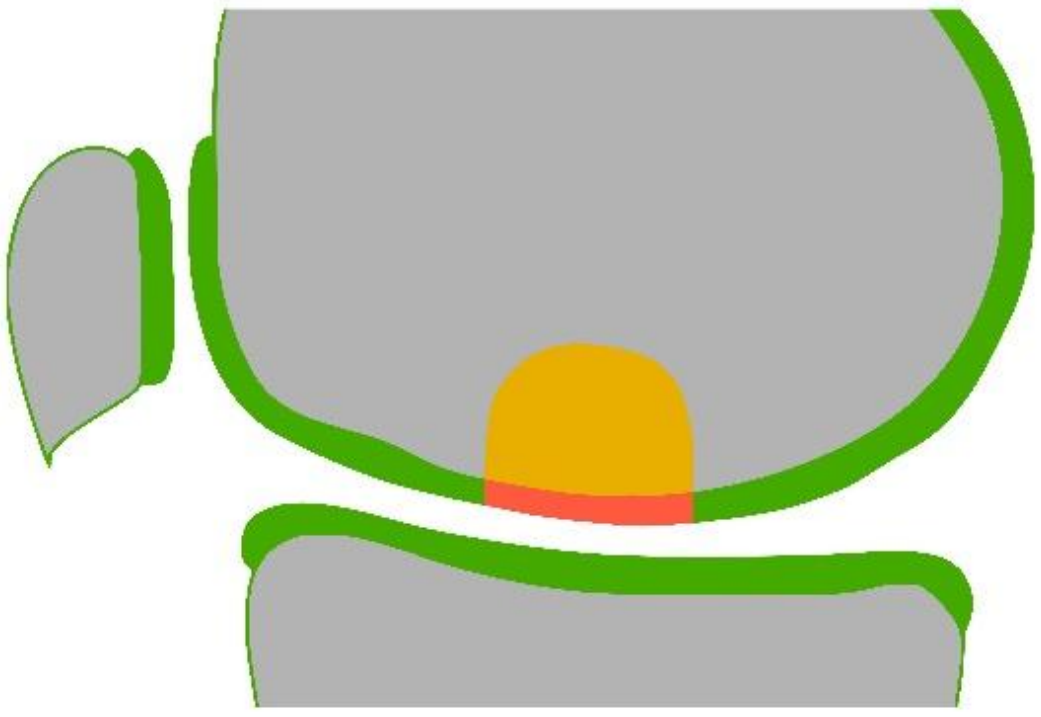

1-A Complete integration (20 pts)

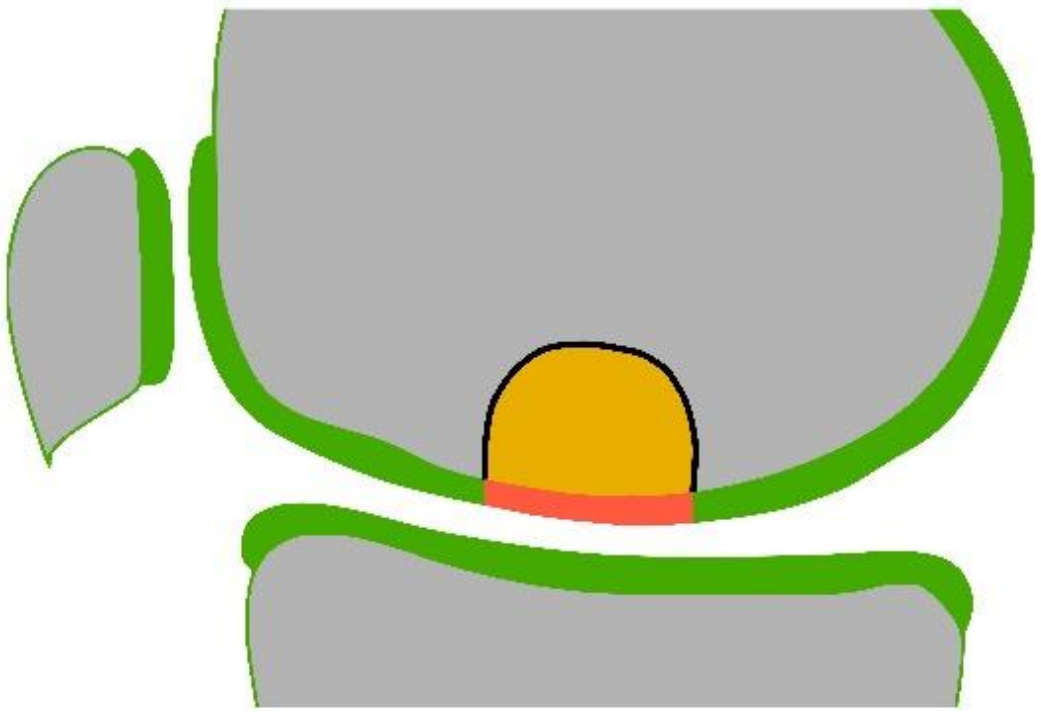

1-B Circumferential or partial demarcating borderline < 2 mm (15 pts)

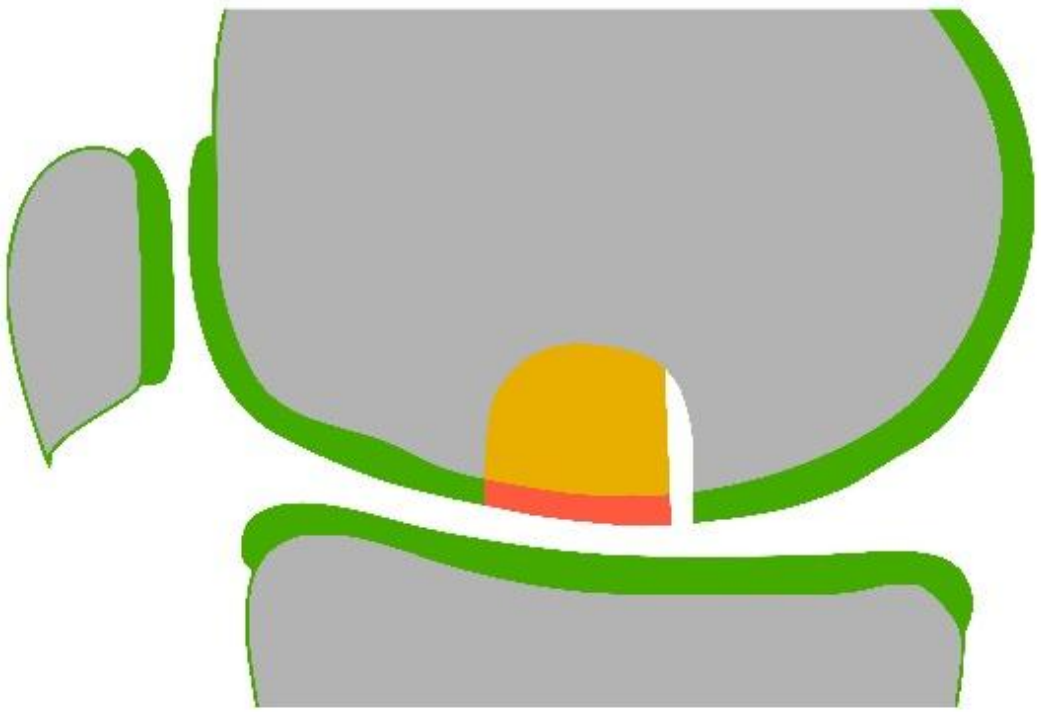

1-C Split-like bony defect < 2 mm at the lateral graft–host interface (fluid-like signal) (10 pts)

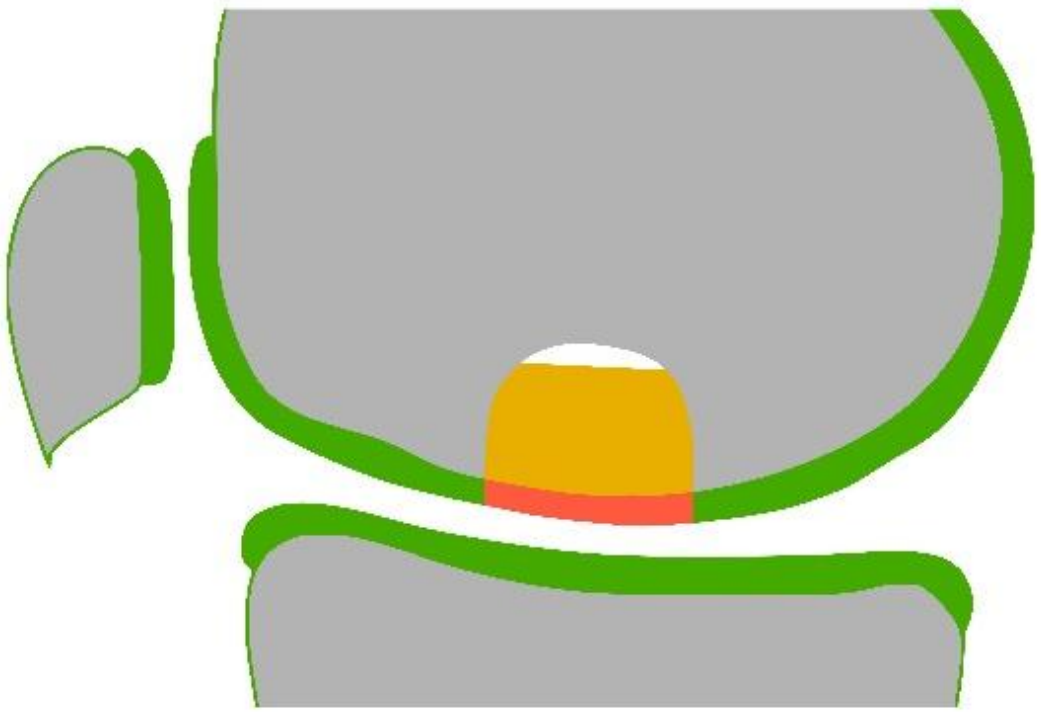

1-D Split-like bony defect < 2 mm at the basal graft–host interface (fluid-like signal) (5 pts)

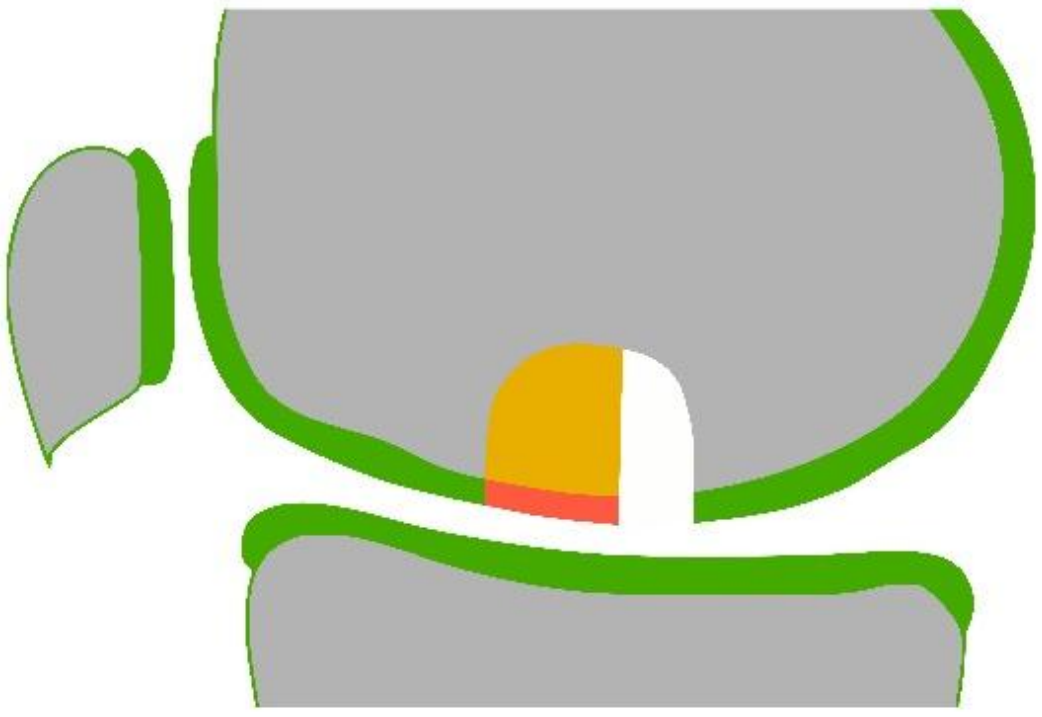

1-E Integration defect > 2 mm (lateral and/or basal graft–host interface) and/or collapsed graft surface (0 pts)

Bone Implant Normalization – 20 points

Assesses structural normalization and remodeling of the implanted osseous component in direct comparison to the surrounding native bone. The focus is on trabecular organization and restoration toward normal bone architecture, rather than on focal subchondral bone marrow edema (captured within MOCART 2.0). Trabecular bone lines are best assessed on non-fat-saturated PD-weighted TSE sequences. A non-osseous marrow pattern within the implant (i.e., absence of trabecular lines) is interpreted as lack of bone normalization. Evaluation is performed relative to the original graft size and configuration. Edema-like signal throughout the implant is defined as involving ≥ 30% of the implant volume with trabecular lines still visible.

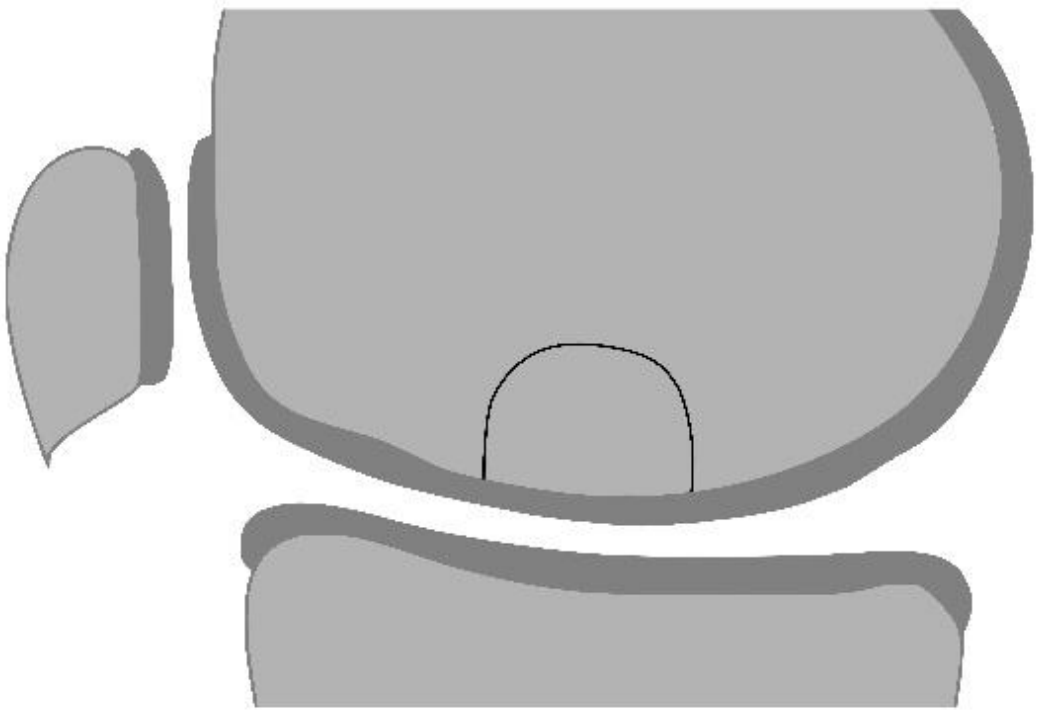

2-A Bone-like marrow signal throughout the implant (20 pts)

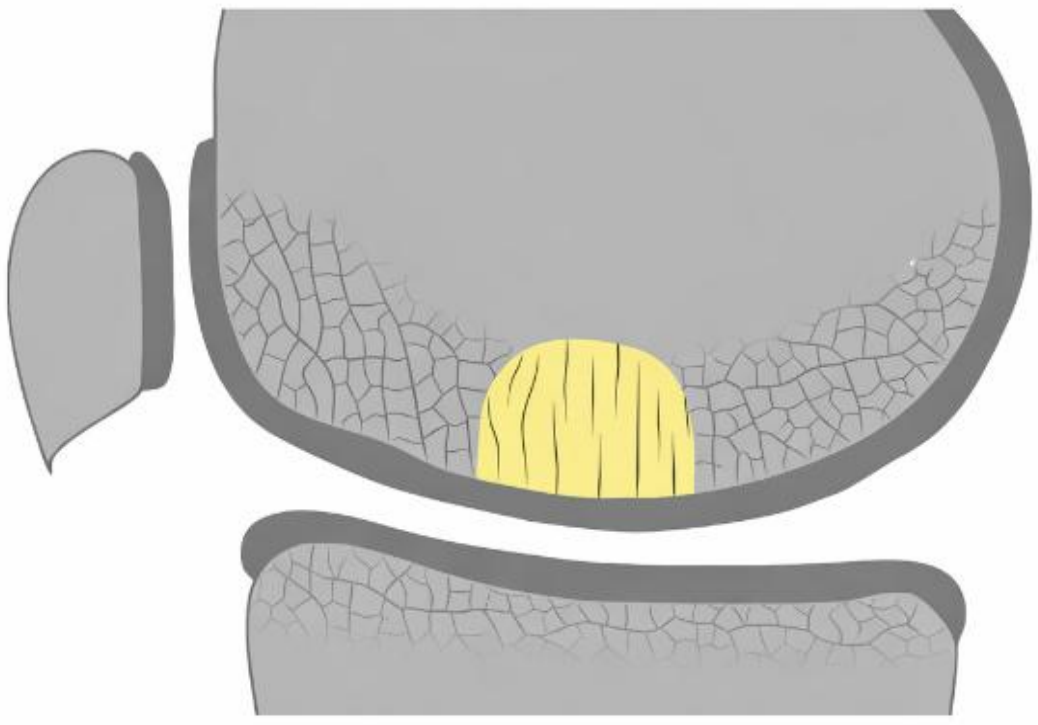

2-B Edema-like signal throughout the implant (≥ 30% of implant volume, trabecular bone lines visible) (15 pts)

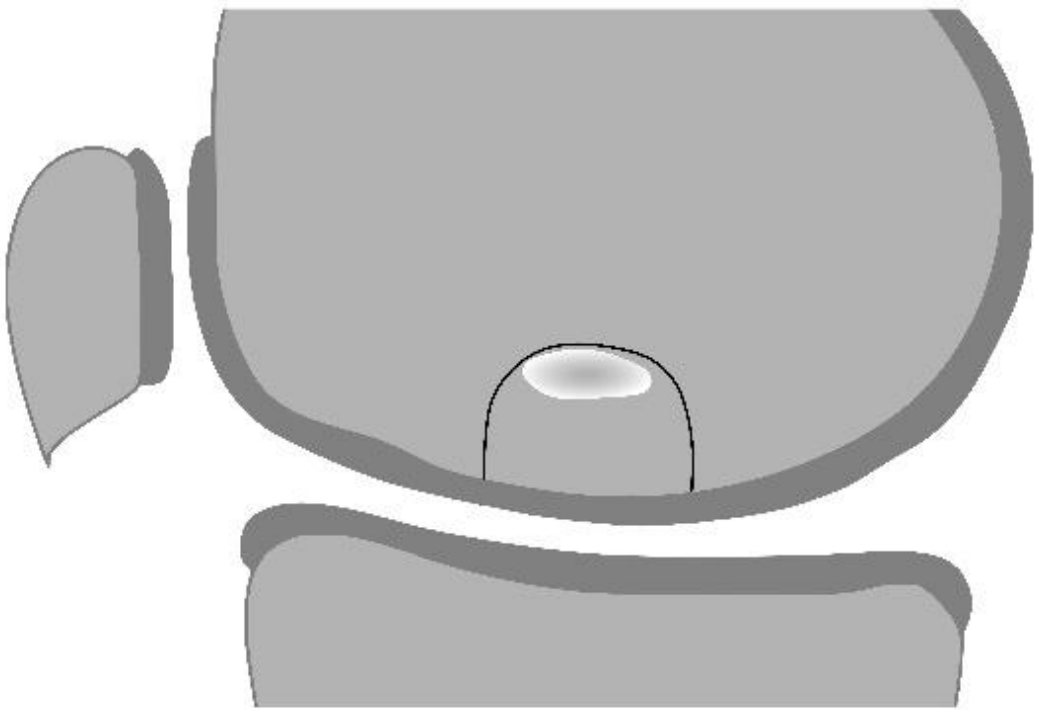

2-C Majority (90–50%) of the implant shows bone normalization (10 pts)

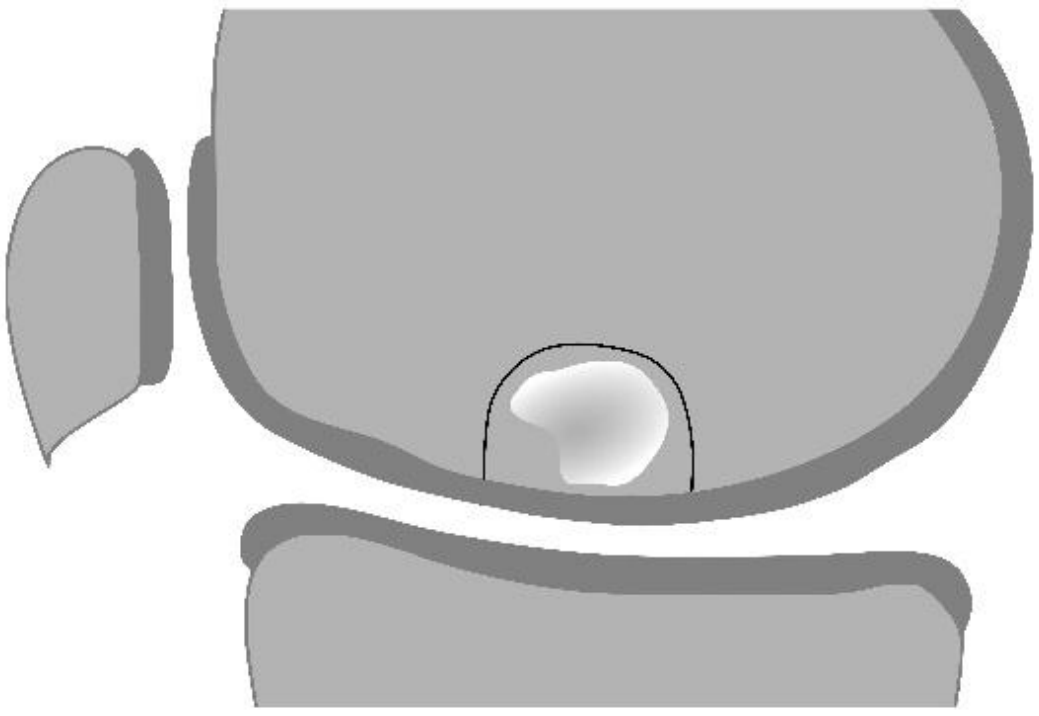

2-D Minority (49–10%) of the implant shows bone normalization (5 pts)

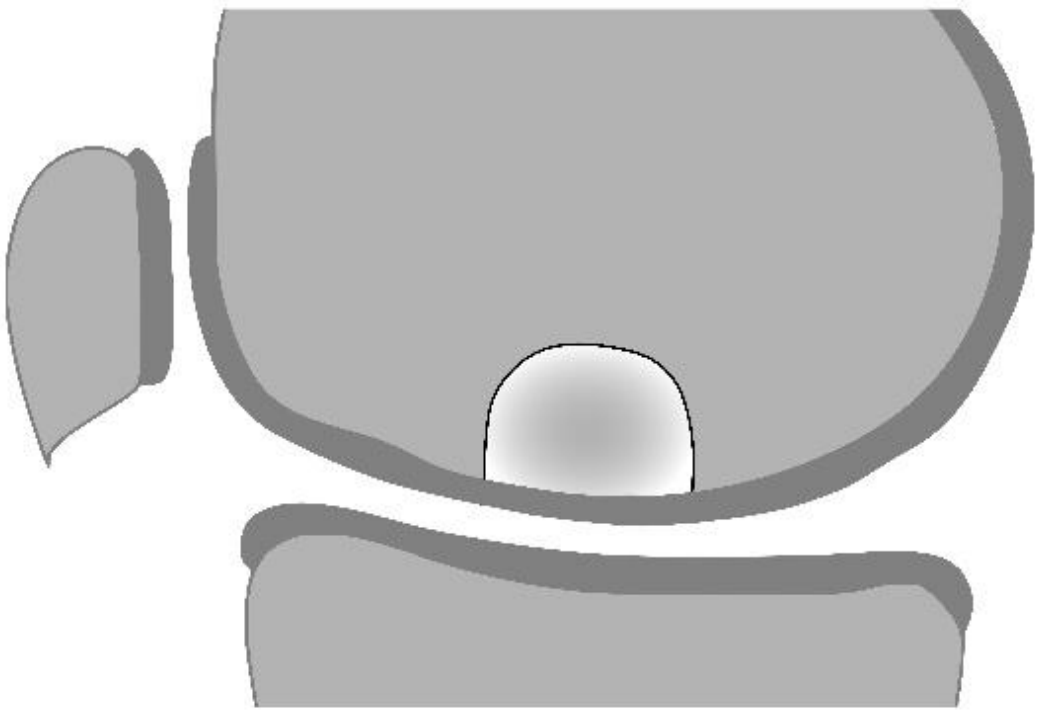

2-E No visible bone normalization (< 10% of implant/scaffold volume) (0 pts)

Bone Implant Cysts – 10 points

Assesses intra-implant cysts within the osseous component that are in direct contact with the implant–host bone interface and/or located within the implant. Cysts are defined as rounded/ovoid, well-demarcated, fluid-like cavities (water-isointense). Cystic lesions must be distinguished from split-like interface defects (integration defects), which present as linear/cleft-like separations along the graft–host interface rather than as a circumscribed cavity. If multiple implant cysts are present, record the combined diameter (sum of the maximal diameters of all cysts within the assessed implant).

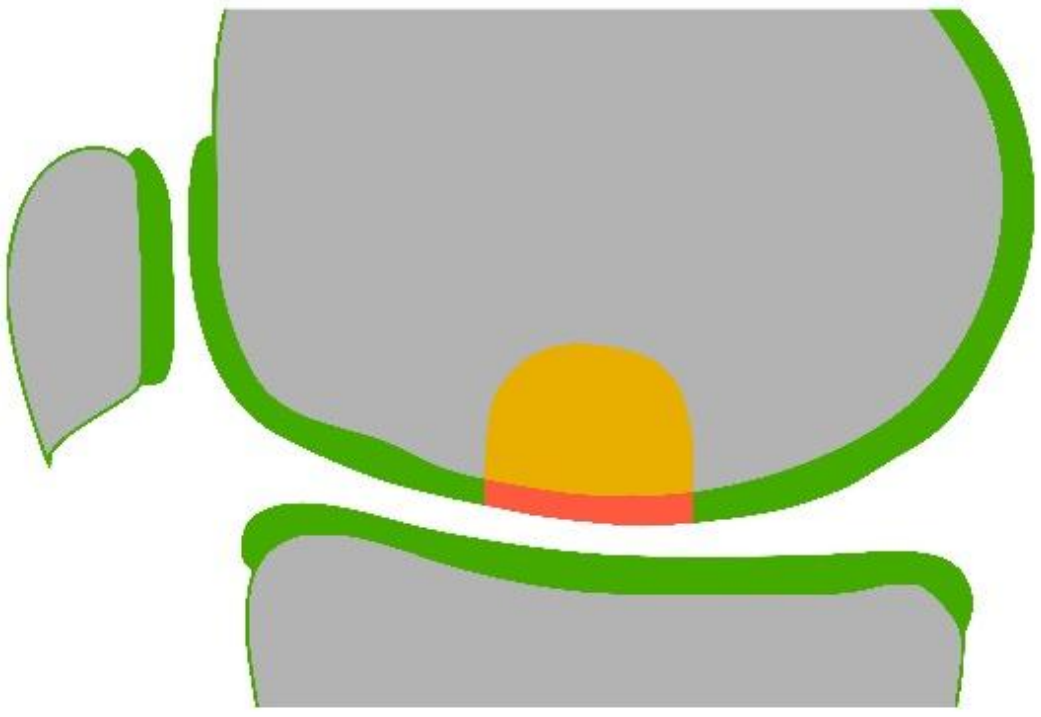

3-A No implant cysts (10 pts)

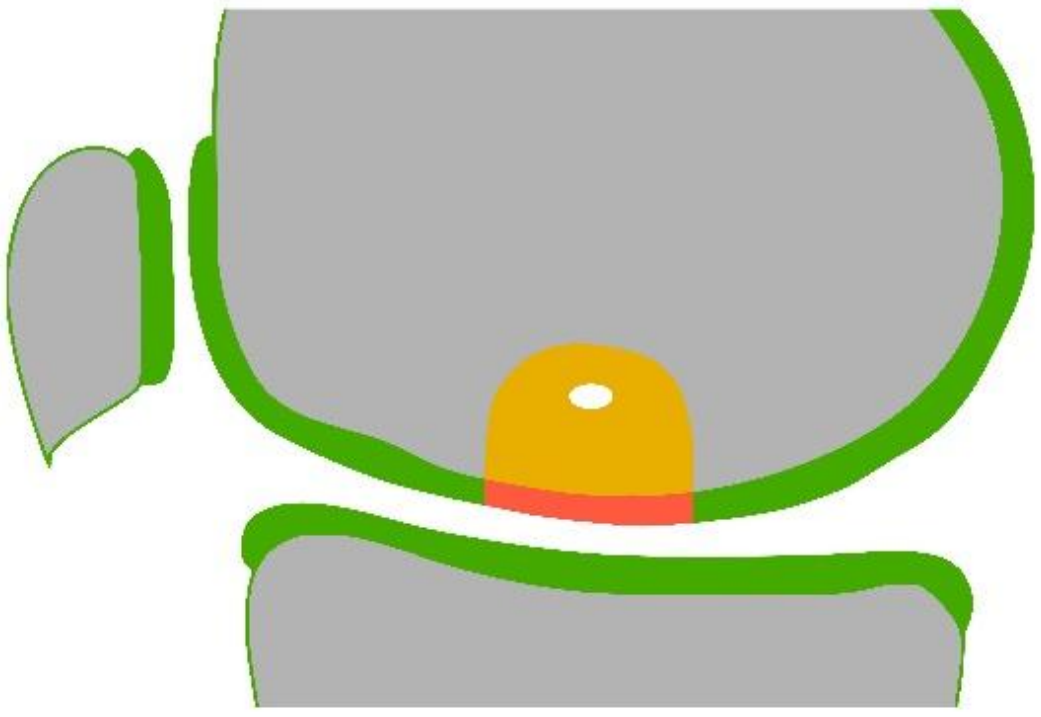

3-B Implant cysts ≤ 5 mm (5 pts)

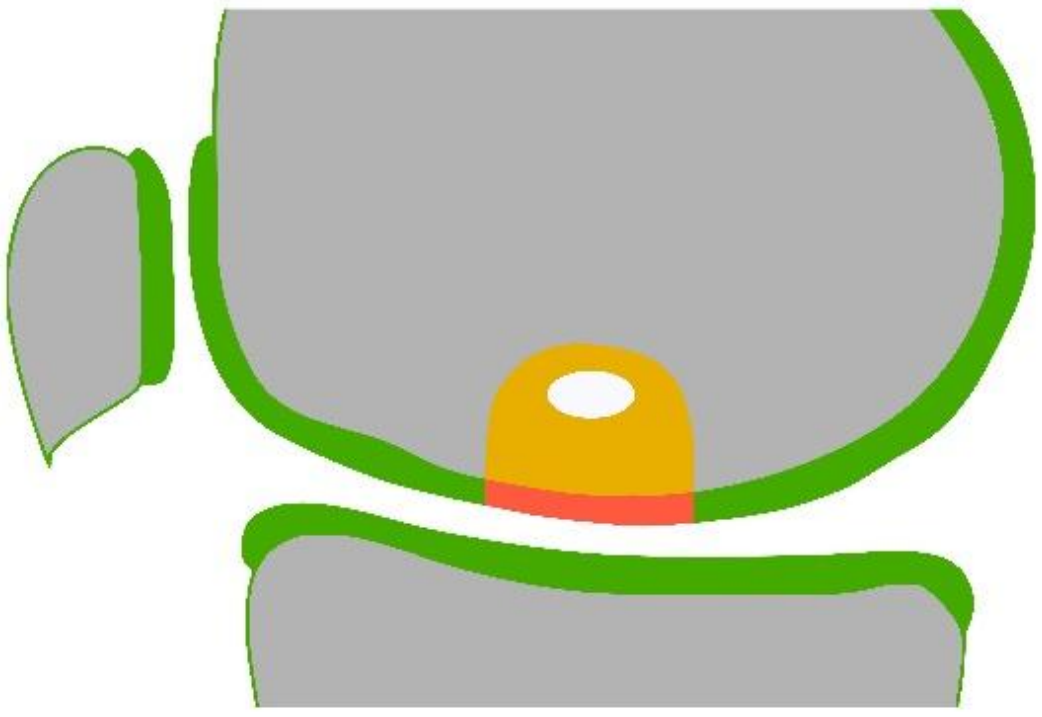

3-C Implant cysts > 5 mm (0 pts)

Reading tips & legend

Color legend

**Green**	Intact native cartilage
**Gray**	Native subchondral bone (and bone-incorporated implant component)
**Orange / yellow filled**	Implanted osseous component (graft / scaffold)
**Red**	Chondral layer of the osteochondral construct at the joint surface (scored within MOCART 2.0, not MOBCART)
**White**	Fluid-like signal: split-like defect, cyst, or non-normalized osseous tissue
**Yellow with trabecular lines**	Edema-like signal with preserved trabecular architecture
**Black borderline**	Demarcating interface line (no fluid-like signal)

Scoring rules

**1 Worst-interface rule (Integration)**	When the lateral/circumferential and basal/deep interfaces show different grades, the worst interface determines the grade for the implant.
**2 Multiple-implant rule**	If multiple repair sites are present in one knee, the lowest-scoring implant determines the final MOBCART add-on score for that case.
**3 Cyst vs split**	A cyst is a circumscribed, rounded/ovoid fluid-like cavity. A split-like defect is a linear/cleft-like separation along the graft–host interface; this is graded under Integration, not Cysts.
**4 Cyst diameter**	With multiple cysts inside one implant, sum the maximal diameters of all included cysts and use the combined diameter for grading.
**5 Boundary to MOCART 2.0**	MOBCART captures intra-implant and implant–host interface findings. Subchondral edema-like marrow signal, subchondral cysts adjacent to the cartilage repair zone, and bony defect/overgrowth in the cartilage repair region are scored within MOCART 2.0, not MOBCART.

Reference: *Koenig FRM, Schreiner M, Szomolanyi P, et al. The MOBCART (magnetic resonance observation of bone and cartilage repair tissue) add-on score. Insights Imaging. 2026. https://doi.org/10.1186/s13244-026-02404-3*

MOCART 2.0 knee score: Schreiner MM, Raudner M, Marlovits S, et al. The MOCART (magnetic resonance observation of cartilage repair tissue) 2.0 knee score and atlas. Cartilage. 2021;13(1 Suppl):571S–587S. https://doi.org/10.1177/1947603519865308
